# Bayesian modeling of post-vaccination serological data suggests that yearly vaccination of dog aged <2 years old is efficient to stop rabies circulation in Cambodia

**DOI:** 10.1371/journal.pntd.0012089

**Published:** 2024-04-18

**Authors:** Heidi Auerswald, Julia Guillebaud, Benoit Durand, Mathilde Le Vu, Sopheak Sorn, Saraden In, Vutha Pov, Holl Davun, Veasna Duong, Sowath Ly, Philippe Dussart, Véronique Chevalier

**Affiliations:** 1 Virology Unit, Institut Pasteur du Cambodge, Pasteur Network, Phnom Penh, Cambodia; 2 Epidemiology Unit, Laboratory for Animal Health, French Agency for Food, Environmental and Occupational Health and Safety (ANSES), University Paris-Est, Maisons-Alfort, France; 3 Epidemiology and Public Health Unit, Institut Pasteur du Cambodge, Pasteur Network, Phnom Penh, Cambodia; 4 General Directorate of Animal Health and Production, Ministry of Agriculture, Forestry and Fisheries, Phnom Penh, Cambodia; 5 Centre de Coopération Internationale en Recherche Agronomique pour le Développement (CIRAD), Unité Mixte de Recherche ASTRE, Montpellier, France; 6 ASTRE, Université de Montpellier, CIRAD, INRAE, Montpellier, France; The University of Sydney School of Veterinary Science, AUSTRALIA

## Abstract

Rabies control remains challenging in low and middle-income countries, mostly due to lack of financial resources, rapid turnover of dog populations and poor accessibility to dogs. Rabies is endemic in Cambodia, where no national rabies vaccination program is implemented. The objective of this study was to assess the short and long-term vaccination-induced immunity in Cambodian dogs under field conditions, and to propose optimized vaccination strategies. A cohort of 351 dogs was followed at regular time points following primary vaccination only (PV) or PV plus single booster (BV). Fluorescent antibody virus neutralization test (FAVNT) was implemented to determine the neutralizing antibody titer against rabies and an individual titer ≥0·5 IU/mL indicated protection. Bayesian modeling was used to evaluate the individual duration of protection against rabies and the efficacy of two different vaccination strategies. Overall, 61% of dogs had a protective immunity one year after PV. In dogs receiving a BV, this protective immunity remained for up to one year after the BV in 95% of dogs. According to the best Bayesian model, a PV conferred a protective immunity in 82% of dogs (95% CI: 75–91%) for a mean duration of 4.7 years, and BV induced a lifelong protective immunity. Annual PV of dogs less than one year old and systematic BV solely of dogs vaccinated the year before would allow to achieve the 70% World Health Organization recommended threshold to control rabies circulation in a dog population in three to five years of implementation depending on dog population dynamics. This vaccination strategy would save up to about a third of vaccine doses, reducing cost and time efforts of mass dog vaccination campaigns. These results can contribute to optimize rabies control measures in Cambodia moving towards the global goal of ending human death from dog-mediated rabies by 2030.

## Author summary

Rabies is a fatal zoonotic viral disease. Dog vaccination is recognized as the most cost-effective and sustainable solution to rabies prevention. Cambodia is endemic with canine mediated rabies, that would cause to around 800 human death each year. This country also has a large owned but mostly free-roaming dog population that makes efficient massive vaccination hard to achieve. In this study, we vaccinated 351 dogs with or without booster one year later. We then used serological assessed the short and long-term vaccination-induced immunity in these dogs, and Bayesian modeling to identify a vaccination strategy adapted to the Cambodian dog population characteristics. Yearly primary vaccinations for young dogs, supplemented with a single booster injection of these dogs promises to achieve 70% dog vaccination coverage effectively and efficiently, and control rabies circulation in dog populations. Applying this new strategy running for five more years after the vaccination coverage of at least 70% is achieved, this implies per 1,000 dogs between 2000 and 5 000 saved vaccine doses depending on dog population characteristics. Our results could help controlling rabies in Cambodia and potentially be adopted by other countries facing similar challenges.

## Introduction

Human infection with rabies virus (RABV) causes almost always fatal encephalitis when not treated by timely administration of the vaccine before the onset of symptoms. It still affects more than 150 countries leading annually to estimated 59,000 deaths, half of them occurring in Asia ([1]. Up to 99% of human rabies cases are acquired from bites of infected dogs. Since dogs are the main reservoir and source of infection for humans, dog vaccination is recognized as the most cost-effective and sustainable solution to rabies prevention [2]. Elimination of rabies in domestic dog populations can be achieved with ≥70% dog vaccination coverage each year for at least five years [3]. Annual vaccination using injectable inactivated virus vaccines is recommended for all dogs irrespective of age and health status. However, vaccination coverage may be limited through insufficient vaccine delivery during vaccination campaigns, the disappearance of vaccinated dogs and/or the introduction of unvaccinated, susceptible dogs through dog movements, trade and demographic processes [4]. The vaccine-induced protection can be compromised by immuno-suppression through malnutrition, infection and other stressors [4,5]. Dog vaccination has been successfully used in many industrialized countries to prevent dog-mediated rabies [3]. However, in low- and middle-income countries (LMICs), controlling and eventually eliminating rabies is more challenging due to the lack of financial resources to sustain vaccination and surveillance over longer time, as well as lack of accessibility to human treatment and dog vaccination especially in rural communities where most rabies cases occur.

Several inactivated canine vaccines are licensed in the EU and US. Most vaccines, available in different formulations, are intended to induce immunity for one to three years [6]. The individual immunization rate induced by vaccination can be monitored by measuring neutralizing antibodies (nAb) as they are a proxy for protection [5]. These antibodies can be measured by virus neutralization assays such as rapid fluorescent focus inhibition test (RFFIT) or fluorescent antibody virus neutralization test (FAVNT) [7]. A nAb titer of ≥ 0·5 IU/mL is considered protective in humans [3] and dogs [7]. The amount of nAbs produced after vaccination can be influenced by individual dog characteristics like age [5,8–10], health status [4,5], sex and neutering status [4,8,10] as well as size and breed [8,9,11,12]. Even vaccination studies of laboratory bred dogs of the same age, breed and health status showed high individual variability of immune reaction and nAb production [5,13].

The factors influencing vaccine-induced protection are complex, and serological profiles after vaccination can oscillate around the binary threshold of nAb titer, often due to inter-assay variability [14–17]. Traditional statistical approaches might not fully capture the variability and interdependencies among dog demographics, vaccination history, and serological responses. The application of latent class models (LCMs) in a Bayesian framework is a strategic choice to assess test sensitivity and specificity in the absence of a gold standard, and Bayesian methods are commonly used to fit this type of modelling [18]. Additionally, these models are well-suited for heterogenous populations where individuals can be categorized into latent groups based on unobservable characteristics. This approach allows us to model the actual immune states of individuals as “latent” states, providing a more nuanced understanding of the underlying serological dynamics.

Rabies is endemic in Cambodia, a Southeast Asian country where around 75% of the people live in rural areas [19]. The dog population in Cambodia is extremely dense, with an estimated dog-to-human ratio of 1:3–4 [20,21]. There is no national rabies vaccination program nor canine population management. It is estimated that annually over 800 people die from rabies but this is likely an underestimation as this calculation is based on data available only for the capital city Phnom Penh and surrounding regions [22]. Like in many endemic countries, financial constraints, the possibility of handling aggressive dogs, and their low survival rate [21], raise questions about the best strategy for feasible and cost-effective annual mass dog vaccinations (MDV).

A multi-partner rabies control program started in 2017 to reduce human rabies cases in the Cambodian provinces Kandal and Battambang. The first achievements of this program were an estimation of demographic parameters of the dog populations, a deep understanding of dog-human relationships and dog management practices, as well as the estimation of the annual bite incidence rate and associated risk factors [21]. Furthermore, this program aimed to design a dog vaccination strategy accounting for the dog demography in Cambodia and the vaccination-induced immunity of these dogs. To achieve this goal, we used post-vaccination serological follow-up accompanying the above-mentioned dog demography survey, and applied Bayesian modeling to evaluate the individual duration of protection against rabies and to compare two different alternative vaccination strategies: annual primary vaccination of young dogs (<1 year old) *vs* annual primary vaccination of young dogs (<1 year old) with systematic booster vaccination solely of dogs that have been vaccinated the year before.

## Materials and methods

### Ethics statement

We followed WOAH guiding principles on animal welfare [23]. The protocol of the survey has been approved by General Directorate of Animal Health and Production of Cambodia (GDAHP). All sampling sessions and interviews were implemented by trained Institut Pasteur du Cambodge officers with supervision of GDAHP agents, local veterinary services and local village authorities. All data were anonymized.

### Vaccination and serological follow-up

The details of the demographic survey performed in Kandal and Battambang provinces are provided in Chevalier *et al*. [21]. During this survey, at enrollment (T0), amenable dogs were vaccinated against rabies via subcutaneous injection of Rabisin (Merial, Lyon, France) provided by the World Organization for Animal Health (WOAH) through the General Directorate of Animal Health and Production of Cambodia (GDAHP). Before, venous blood was collected either from jugular or brachial vein, serum was separated from the clot by centrifugation and then was stored at -20°C until serological analysis.

**Fig 1:**
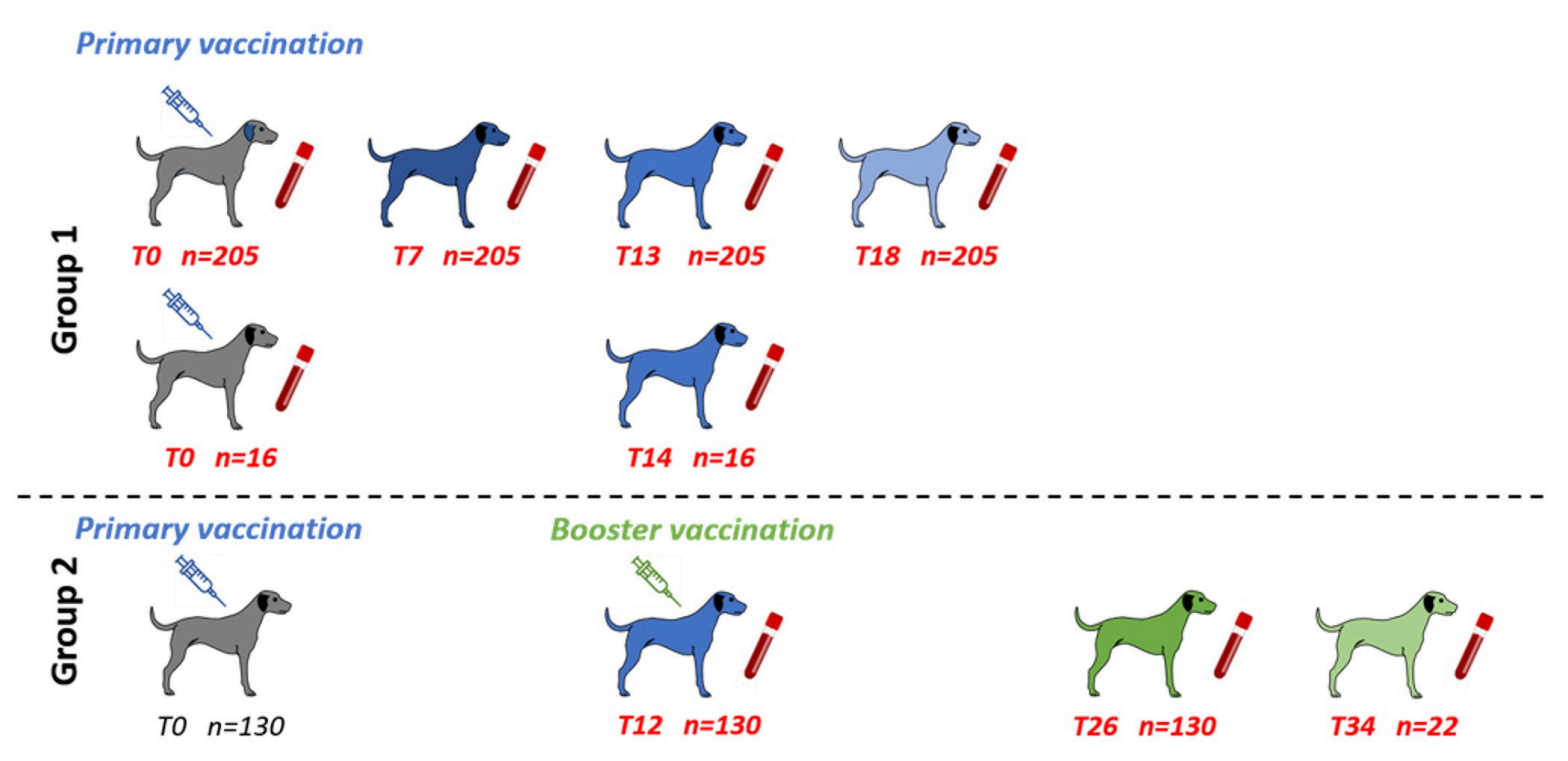
Vaccination and follow-up schedule for study groups (created by authors using the licensed program BioRender (http://www.biorender.com). Dogs without documented previous rabies vaccination (grey) received their primary vaccination (T0, grey). For group 1 follow-up blood samples were taken seven months (T7), around one year (T12-14) and 1.5 years (T18) after primary vaccination. Due to logistic restrains the follow-up sequence differs (blue) within this group between dogs from Kandal (n = 16) and dogs from Battambang (n = 205). From dogs of group 2, a blood sample (T12) was collected one year after their primary immunization to document the immune response of this primary immunization. Afterwards these dogs received a booster vaccination (green), and additional samples were collected (green) more than one year (T26) and three years (T34) after the booster vaccination to monitor its effect on the immune response.

The study included two groups of dogs (Fig 1). Group 1 (n = 221) relates to dogs with an individual blood sample at enrollment (T0) before these dogs received their primary vaccination. These dogs were formally re-identified with their owners, and re-sampled approximately every 6 months for up to 1.5 years (18 months, T18). Dogs of group 2 (n = 130) were primary vaccinated (T0) without a prior blood collection. These dogs were re-captured and re-identified one year after their primary vaccination, a blood sample was collected and they received a booster vaccination (T12). An additional follow-up blood collection of group 2 dogs was performed 14 months (T26, n = 130), and 22 months (T34, n = 22) after booster vaccination.

### Fluorescent antibody virus neutralization test (FAVNT)

FAVNT was used to determine RABV nAb titer in dogs at T0, T6, T12, T18, T24 and T36. The assay was performed following WOHA guidelines [7,24]. Antibody titers of ≥ 0·5 IU/mL were considered positive [25].

### Statistical model

Age of the dogs at primary vaccination was categorized in four groups: <12 months, 12–24 months, 24–48 months and >48 months old based on the 25^th^, 50^th^ and 75^th^ percentiles of the age distribution. The body condition score (BCS) was categorized as “skinny” for BCS<4, “normal weight” for BCS 4–6, and “overweight” for BCS>6.

**Fig 2:**
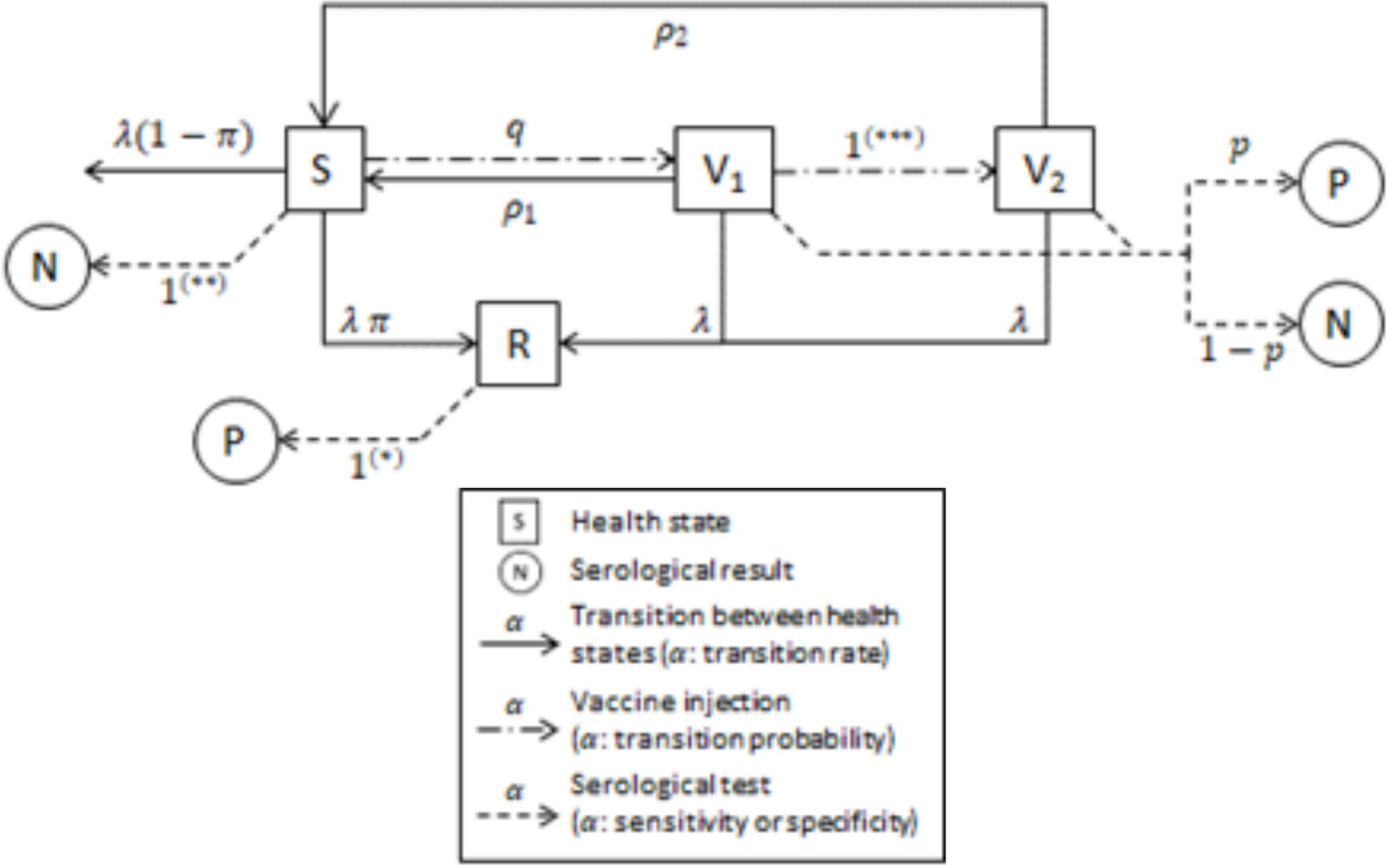
Probabilistic model of the evolution of dog rabies and serological status during the study period. The whole dog population is separated in 4 states (indicated by squares): *S* susceptible dog; *V*_*1*_ dog with protective immunity induced by primary vaccination; *V*_*2*_ dog with protective immunity induced by primary and subsequent booster vaccination; *R* dog with protective immunity induced by a non-lethal infection. The serological results (by FAVNT) are indicated by circles: *N* negative FAVNT (<0·5 IU/mL); *P* positive FAVNT (≥0·5 IU/mL). The parameters describing the model dynamics are: *λ* average force of infection; *π* probability of non-lethal infection; *q* probability of acquiring protective immunity after vaccination; *ρ*_*1*_ rate of loss of protective immunity induced by primary vaccination; *ρ*_*2*_ rate of loss of protective immunity induced by primary and subsequent booster vaccination; *p* sensitivity of the serological test for dogs in *V*_*1*_ or *V*_*2*_ status. (*) Sensitivity is assumed perfect for animals in the R state. (**) Specificity is assumed perfect. (***) Dogs in the *V*_*1*_ state are assumed immuno-competent (since they have acquired a protective immunity after primary vaccination), therefore, all are assumed to enter the *V*_*2*_ state after the booster vaccination.

Subsequently, the term of ‘protective immunity’ is used to refer to an immune state indicated by a positive FAVNT result of ≥0.5 IU/mL as this is indicated by the World Organization for Animal Health as a reasonable level of seroconversion after rabies vaccination [26]. We modeled the immune state of each dog in the study cohorts using three states: susceptible (denoted S), having acquired a protective immunity after a primary vaccination (denoted V_1_), or after a booster vaccination (denoted V_2_). As RABV is endemic in Cambodia, some dogs could have been naturally exposed to RABV prior to, or during the study, and therefore have developed a non-lethal infection and subsequent immunity. This may bias the analysis. To control for this bias, we explicitly represented natural exposure to RABV in the model, and added a 4^th^ immune state for dogs that acquired a protective immunity after a natural infection (denoted R). In order to create a usable, simplified model we assumed that in this latter case, the protective immunity was lifelong. Transitions between immune states are described in Fig 2. Vaccination of a susceptible dog (naïve, or with lost protective immunity after primary or booster vaccination) induced a protective immunity (state V1) with a probability q. The protective immunity is subsequently lost with a rate ρ1. We assume that the dogs experience a constant rate of immunity loss post-vaccination, suggesting that the duration of immunity follows an exponential distribution. This hypothesis is particularly relevant in the Cambodian context, given the short life expectancy of dogs there. We assumed that dogs that received a booster vaccination (state V2), while already protected since primary vaccination, lost their protective immunity with a different rate (ρ2). All dogs were assumed exposed to RABV via natural infection (or to a rabies-like lyssavirus inducing a cross-protection) with a force of infection λ. When exposed, susceptible dogs may develop a non-lethal form with a probability π (or died otherwise with a probability of 1-π). They then acquired lifelong protective immunity (R state). We assumed that FAVNT allowed the detection of protective immunity (nAb titer ≥ 0·5 IU/mL) in vaccinated dogs (states V1 and V2) with a probability p, and that dogs that acquired a natural immunity (R state) were always positive. The specificity of the FAVNT was assumed perfect.

The dynamics of the different immune states in a cohort of dogs receiving a primary vaccination at *t = PV*, and a booster vaccination at *t = BV*, is described by the following equations (with 1_*b*_ the dummy function: *1*_*b*_ *= 1* if *b* is true, and 0 otherwise):

$$\frac{dS}{dt}=-\lambda S-1_{\left(t=PV)or(t=BV\right)}qS+\rho_{1}V_{1}+\rho_{2}V_{2}$$

$$\frac{dV_{1}}{dt}=1_{\left(t=PV)or(t=BV\right)}qS-V_{1}(\rho_{1}+\lambda )-1_{t=BV}V_{1}$$

$$\frac{dV_{2}}{dt}=1_{t=BV}V_{1}-V_{2}(\rho_{2}+\lambda )$$

$$\frac{dR}{dt}=\lambda (\pi S+V_{1}+V_{2})$$

The model was not identifiable since *λ* and *π* always appeared in a multiplicative way. We therefore defined three scenarios for *π*, the probability to acquire/develop a non-lethal infection: the estimate of 0·51 proposed by Hampson *et al*. [27], and two arbitrary extreme values of 0·05 and 0·95. The five remaining parameters of the model (*q*, *λ*, *ρ*_1_, *ρ*_2_ and *p*) could then be estimated for each scenario. We analyzed the influence of three individual dog characteristics (age group, BCS, and sex) on the five model parameters. For a given dog characteristic and a given parameter, we compared the model in which the parameter changed according to the dog characteristic, with the reference model in which the respective parameter value was the same for all dogs. We thus successively compared 15 alternative models (five parameters × three individual characteristics) with the reference model, using the Bayes’ factor, interpreted according to the usual rules: the strength of evidence in favor of the alternative model (relative to the reference model) was considered substantial if 3<BF<20, strong if 20<BF<150, and very strong if BF>150. Models were fitted in a Bayesian Markov Chain Monte Carlo (MCMC) framework. Priors were non-informative. We used R version 4.2.1 [28] and RStan version 2.21.5 [29]. R and Stancodes, and raw data- group1a (n = 205), group1b (n = 16) and group2 (n = 130) are provided in S1 Text.

### Evaluation of vaccination strategies

We used the best model estimates to compare two vaccination strategies: (A) annual primary vaccination of dogs <1 year old; (B) annual primary vaccination of dogs <1 year old with systematic booster vaccination of dogs that have been vaccinated the year before. For each of the strategies, we calculated the percentage of the protected population *N* years after the strategy started. In this calculation, we considered only vaccine-induced immunity, and neglected natural immunity (induced by non-fatal infection or cross-protection due to infection with rabies-like lyssavirus). Mortality rates for each dog population was assumed constant for this preliminary analysis. Details on calculations are provided in S2 Text.

We calculated the proportion of the dogs with vaccine-induced protective immunity each year during the first ten years, and after 20 years of implementation (*i*.*e*. after complete renewal of the dog population). Two different Cambodian dog populations were considered: Kandal province with a 24-months survival rate of 52%, and Battambang province with 34% as described previously [21]. Confidence intervals (CIs) were calculated using the lower limit of the CI of *q* and the upper limit of that of *ρ*_1_ and *ρ*_2_, for the lower bound, and the upper limit of the CI of *q* and the lower CI limit of that of *ρ*_1_ and *ρ*_2_ for the upper bound.

## Results

### Cohort characteristics

In total, we included 351 dogs, where 221 dogs received a primary vaccination (group 1), and 130 dogs received an additional booster vaccination (group 2). The median age of dogs at inclusion was 24 months (min = 1 months; max = 17 years). Overall, 78% (273/351) of the dogs had a medium BCS, corresponding to normal weight. The male-to-female ratio was 1·26.

### Serological results

Serological results were used to categorize each dog into different trajectory groups regarding their serological status dynamics (Table 1)

**Table 1:** Dynamics of serological status.

Primary vaccination*							**Number of dogs**
**Time points**	**T0**	**T7**	**T12-T14**	**T18**	**T26**	**T34**	
Trajectories of serological status	N	N	N	P	n/a	n/a	5
	N	N	P	N	n/a	n/a	7
	N	N	P	P	n/a	n/a	7
	N	P	N	N	n/a	n/a	14
	N	P	N	P	n/a	n/a	4
	N	P	P	N	n/a	n/a	19
	N	P	P	P	n/a	n/a	85
	P	P	P	P	n/a	n/a	5
	N	n/a	N	n/a	n/a	n/a	4
	N	n/a	P	n/a	n/a	n/a	10
	P	n/a	P	n/a	n/a	n/a	2
Seropositive dogs	3.3% (7/211)	62.0% (127/205)	61.1% (135/221)	51.7% (106/205)	n/a	n/a	
Booster vaccination**							**Number of dogs**
**Time points**	**T0**	**T7**	**T12-T14**	**T18**	**T26**	**T34**	
Trajectories of serological status	n/a	n/a	N	n/a	N	n/a	6
	n/a	n/a	N	n/a	P	n/a	34
	n/a	n/a	N	n/a	P	P	10
	n/a	n/a	P	n/a	N	n/a	1
	n/a	n/a	P	n/a	P	n/a	67
	n/a	n/a	P	n/a	P	P	12
Seropositive dogs	n/a	n/a	61.5% (80/130)	n/a	94.6% (123/130)	100.0% (22/22)	

* Primary rabies vaccination on T0

** primary rabies vaccination on T0 and booster vaccination on T12. N negative FAVNT (<0·5 IU/mL); P positive FAVNT (≥0·5 IU/mL). n/a indicates not applicable as no sample collection was performed on the respective time point

Overall, 61% of the dogs had a protective immunity one year after their primary vaccination. In the dogs receiving a booster vaccination, the rate of protected dogs rose to 95% measured one year after booster vaccination. Seven dogs presented positive nAb titers (≥ 0·5 IU/mL) before they received the rabies vaccination. Overall, 29% (n = 59) remained seronegative after primary vaccination. Eighty-five dogs (41%) remained seropositive through T6 and T18, whereas 9% (n = 19) were positive at T6 but became negative at T18, and 7% (n = 14) were positive at T6 but negative at T12. Nineteen dogs were negative at T6, but became positive at T12 (n = 14) or T18 (n = 5) without being re-vaccinated. For the dogs that received a booster vaccination (group 2, n = 130) and were already seropositive T12 (n = 80), one turned negative at T24, and 67 (84%) remained positive at T24. Among dogs that were negative at T12 (n = 50), 6 were still negative at T24. In the small cohort of dogs (n = 22) that were follow up until two years after their booster vaccination (three years after primary vaccination, T36) 100% of dogs remained positive over the whole study period.

### Modeling of transmission and immunity dynamics

Estimated Bayes’ factor in favor of the alternative models (in which one of the four estimated parameters varies with one of the three dog characteristics) versus the reference model (with all parameters having the same value for all dogs) was always <3 (Table 2). We thus selected the reference model for further analysis.

**Table 2:** Bayes’ factor based comparison of 15 alternative models under three scenarios of non-lethal infection probability upon exposure to RABV.

Estimated parameter	Dog characteristic	Proportion of non-lethal infections		
		*π* = 0·51	*π* = 0·05	*π* = 0·95
*q*	Age group^a^	0·01	0·02	0·01
	Body condition^b^	0·20	0·22	0·20
	Sex	0·30	0·35	0·30
*λ*	Age group^a^	0·07	<0·01	<0·01
	Body condition^b^	<0·01	<0·01	<0·01
	Sex	<0·01	0·01	<0·01
*ρ*_1_	Age group^a^	<0·01	<0·01	<0·01
	Body condition^b^	<0·01	<0·01	<0·01
	Sex	0·16	0·21	0·16
*ρ*_2_	Age group^a^	<0·01	<0·01	<0·01
	Body condition^b^	<0·01	<0·01	<0·01
	Sex	<0·01	<0·01	<0·01
*p*	Age group^a^	<0·01	<0·01	<0·01
	Body condition^b^	0·03	0·05	0·03
	Sex	0·11	0·14	0·11

*π* probability of non-lethal infection upon exposure; *q* probability of acquiring protective immunity after vaccination; *λ* average force of infection exerted on dogs before and during the study period; *ρ*_1_ rate of loss of the protective immunity induced by primary vaccination; *ρ*_2_ rate of loss of the protective immunity induced by primary and booster vaccination; *p* sensitivity of the serological test for dogs in the V_1_ and V_2_ states. ^a^ Four age groups based on quantiles of age distribution: age < 12 months, 12–23 months, 24–48 months. ^b^ Three classes of body condition score (BCS): skinny (BCS ≤3), normal (BCS 4–6) and overweight (BCS >6). In the reference model, parameter values do not depend on dog characteristics. Bayes’ factor (BF) was interpreted according to the usual rules: the strength of evidence in favor of the alternative model (relative to the reference model) is substantial if 3< BF <20, strong if 20< BF <150, and very strong if BF >150

According to the selected model, primary vaccination conferred a protective immunity in 82% of dogs (95% CI: 75–91%), for a mean duration of 1/ρ1 = 4·7 years (95% CI: 3·1–8·2 years). Booster vaccination induced a lifelong protective immunity (the lower bound of the credibility interval was 21 years). Both results were only marginally affected by changes of the value for the proportion of non-lethal infections (Table 3). As expected, the proportion of non-lethal infections affected the estimated force of infection.

**Table 3:** Estimated parameter values obtained using the selected model for three scenarios of the probability of non-lethal infection upon exposure to RABV.

Parameter	Unit	Proportion of non-lethal infections		
		*π* = 0·51	*π* = 0·05	*π* = 0·95
*q*	Probability	0·82^a^ (0·75–0·91)^b^	0·84 (0·75–0·94)	0·83 (0·75–0·91)
*λ*	month^-1^	0·003 (0·001–0·004)	0·016 (0·010–0·022)	0·002 (0·001–0·003)
*ρ*_1_	month^-1^	0·018 (0·010–0·027)	0·022 (0·013–0·035)	0·017 (0·010–0·026)
*ρ*_2_	month^-1^	0·003 (0·001–0·005)	0·002 (0·000–0·006)	0·001 (0·000–0·004)
*p*	Probability	0·90 (0·79–0·97)	0·87 (0·82–0·91)	0·91 (0·87–0·94)

*π* probability of non-lethal infection upon exposure; *q* probability of acquiring protective immunity after vaccination; *λ* average force of infection exerted on dogs before and during the study period;*ρ*_1_ rate of loss of the protective immunity induced by primary vaccination;*ρ*_2_ rate of loss of the protective immunity induced by primo- and booster vaccination; *p* sensitivity of the serological test for dogs in the V_1_ and V_2_ states. ^a^ Mean of the posterior distribution. ^b^ 95% credibility interval.

### Evaluation of vaccination strategies

**Fig 3:**
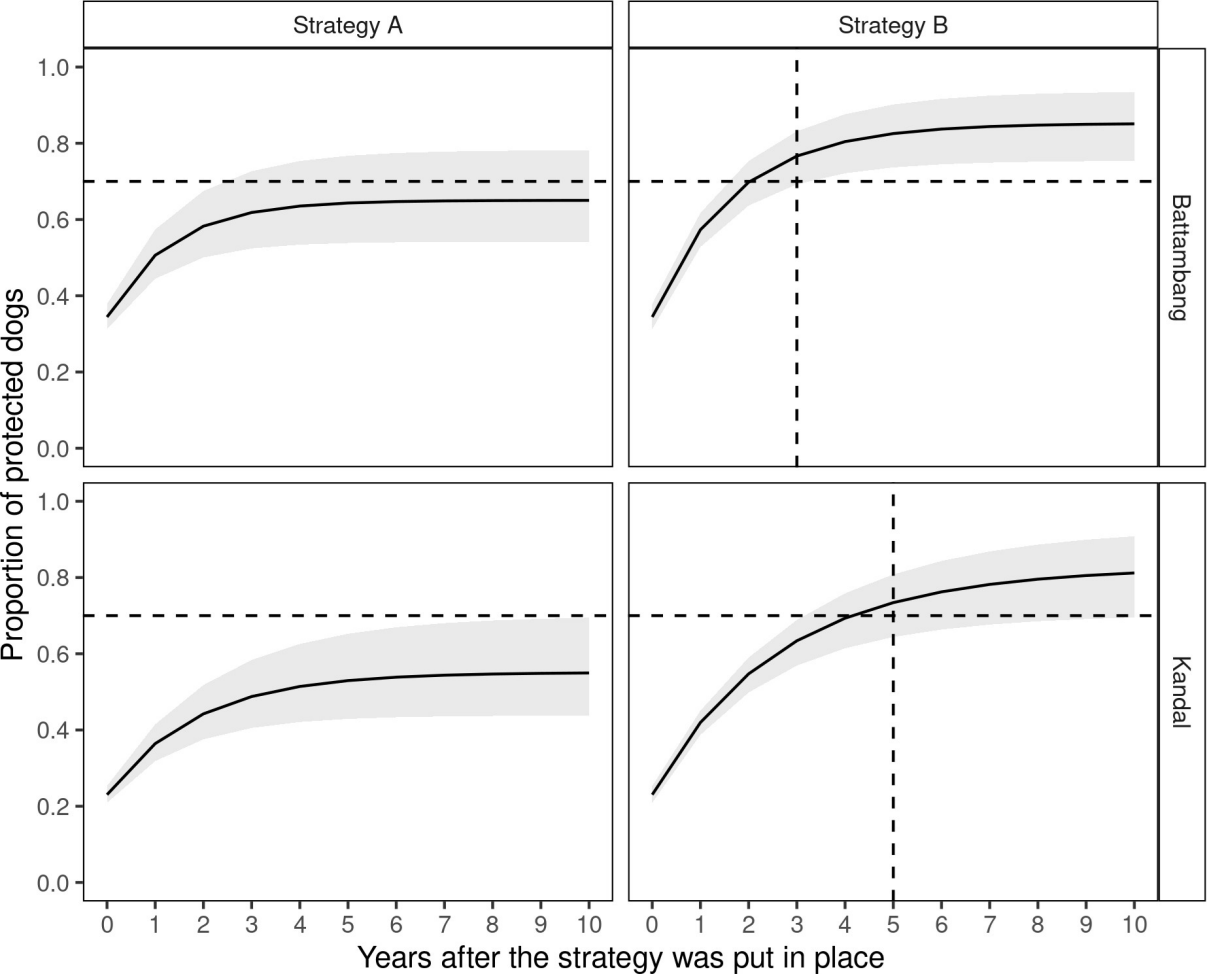
Evolution of the proportion of protected dogs in two Cambodian provinces. For the dog population of the Cambodian provinces of Kandal and Battambang, the proportion of protected dogs were modeled for two vaccination strategies: (A) annual primary vaccination of animals <1 year old and, (B) annual primary vaccination of animals <1 year old and systematic booster vaccination of dogs that have been vaccinated the year before. Plain lines indicate the mean value and grey areas show the 95% confidence intervals. Horizontal dashed lines mark the threshold of 70% vaccination rate recommended by WOAH to control rabies circulation in a dog population, and the vertical dashed lines indicate the year at which this threshold is achieved after implementation of the strategy.

Following vaccination strategy A, 20 years after its implementation with an annual primary vaccination of 100% of the dogs <1year old, the proportion of protected dogs was 55% (95% CI: 44–70) and 65% (95% CI: 64–78) in Kandal and Battambang, respectively (Fig 3). With vaccination strategy B (annual primary vaccination of <1 year old dogs with a systematic booster vaccination of dogs that had been vaccinated the year before), the proportion of dogs with protective immunity would reach 83% in Kandal (95% CI: 70–93%) and 85% in Battambang (95% CI: 75–94%). The recommended 70% vaccination coverage would be achieved in Battambang three years after implementation of this vaccination strategy, and in Kandal after 5 years (Fig 3).

To calculate the vaccine doses that could be saved when our proposed second strategy would be applied instead of annual vaccination of all dogs, we assumed an exponentially distributed life expectancy. For a vaccination campaign applying this new strategy running for five more years after the vaccination coverage of at least 70% is achieved, this implies per 1,000 dogs an amount of 5,200 saved vaccine doses for the Kandal dog population and 2,720 doses when applied in Battambang province (Table 4).

**Table 4:** Estimation of vaccine doses for different vaccination strategies in Kandal and Battambang province.

	Kandal	Battambang
Duration (years) of strategy B*	10	8
**Necessary vaccine doses per 1,000 dogs**		
**Strategy B**	4,800	5,280
**Annual vaccination**	10,000	8,000
**Saved doses**	5,200	2,720

* Vaccination program for five more years after at least 70% vaccination coverage was reached

## Discussion

We used a post-vaccination serological follow-up to investigate the development and persistence of RABV nAbs in 351 dogs under field conditions in Cambodia after a single Rabisin vaccination, with or without booster injection one year after primary vaccination. We then used Bayesian modelling to compare immunization coverage obtained using two different vaccination strategies: (A) annual primary vaccination of animals <1 year old and, (B) annual primary vaccination of animals <1 year old with a systematic booster vaccination of dogs that have been vaccinated the year before.

The peak of antibody response after primo-vaccination is expected 2–3 weeks after vaccination [5,30,31]. Due to logistical constraints, this recommended sampling could not be achieved. However, our results are in concurrence with other studies even if findings of other studies vary greatly (Table 5). One year after the primary vaccination we observed a protective nAb titer in 61% of the dogs. Failure to induce protective immunity with single injection in our study appeared high, with 29% (n = 59) of dogs that received primary vaccination remained seronegative until T18. However, loss of immunity is probably overestimated as dogs were sampled the earliest six months after vaccination and therefore the seroconversion rate usually determined 2–3 weeks after vaccination could not be measured timely. Additionally, vaccinated dogs with a low or negative nAb level may still be protected because of cellular immunity triggered by primary vaccination [5,32].

**Table 5:** Studies on the immunogenicity of rabies field primary vaccinations.

Study site	Vaccine	Method used for serology	Number of tested dogs	Duration of study	Observed seroprevalence	Reference
**Peru**	Rabisin	RFFIT	198	1 year	97%	[49]
**Finland**	Madivak	RFFIT	47	1 year	91%	[50]
**Finland**	Rabisin	RFFIT	85	1 year	84%	[50]
**Tunisia**	Rabisin	RFFIT	200	1 year	73%	[51]
**Thailand**	Rabdomun	RFFIT	31	1 year	72%	[52]
**Cambodia**	Rabisin	FAVNT	221	1 year	61%	this study
**Japan**	Various*	RFFIT	92	1 year	51%	[53]
**Sri Lanka**	Nobivac	RFFIT	110	1 year	50%	[54]
**Indonesia**	Rabisin	ELISA (Pusvetma, Indonesia)	171	90 days		[55]

* 6 different commercially available rabies vaccines, all derived from the RC-HL strain

Measuring rabies nAbs is only a proxy for protection and might not reflect the whole spectrum of the immune response and protection upon a rabies vaccination. Besides humoral immunity, vaccination induces immunologic memory even in dogs with a nAb titer < 0·1 IU/mL as seen in laboratory RABV challenge [32].

In our study, seven dogs presented positive nAb levels (≥ 0·5 IU/mL) before they received RABV vaccination. Moreover, 19 dogs were seronegative at T6, and became positive at T12 (n = 14) or T18 (n = 5) without being re-vaccinated. Vaccine-unrelated rise in nAb titers or the presence of nAb before primary vaccination is well documented [30,33–36] and can be explained either by (i) maternal immunity, (ii) non-lethal exposure and/or (iii) cross-reaction due to exposure with related lyssaviruses. The presence of maternal nAbs is highly unlikely in our study due to the age of most of the included dogs (>3 months) and the fact that rabies animal vaccination is not accessible in rural Cambodia. Maternal antibodies are unlikely to protect the pups longer than six weeks after birth [37]. Several RABV challenge trials had the issue of insufficiently virulent virus strains [32,38], building evidence for non-lethal RABV exposure. Our dogs with nAb prior to rabies vaccination could also have been exposed to a non-rabies bat lyssavirus, widely distributed in South-East Asia [39–41], as some of them are known to cross-neutralize canine RABV [42,43].

Several dogs presented unexpected serological profiles oscillating between seropositive and–negative results. Several explanations can be put forward (vaccination failure, non-lethal infection, lack of sensitivity of the test) but confirm again that a positive result allows to identify a protected dog but that a negative result does not allow to conclude that the dog is susceptible. The fact that the serological test does not allow to detect 100% of protected dogs was considered a lack of sensitivity in the Bayesian model. This model therefore allowed (i) to explicitly represent the non-observable biological status of dogs and (ii) to evaluate the sensitivity of serological testing. According to the model, the mean duration of immunity conferred by primary vaccination was 4·7 years for 82% of vaccinated dogs. A booster injection one year later allowed to induce a protective immunity when the previous injection failed, and to induce a lifelong protective immunity in dogs already seropositive after primary vaccination. Our models’ predictions extend beyond the observed data timeframe. The short life expectancy of dogs in Cambodia justifies our assumption of a constant rate of immunity loss post-vaccination, leading to an exponential distribution for the immunity duration. This assumption might not hold in other contexts where dogs experience longer life expectancies. Therefore, validating this hypothesis with more extended follow-up studies is crucial for broader application. However, the estimated duration of immunity in our study aligns with findings from previous studies. For instance, a challenge trial showed that 80% of dogs survived more than 6 years post-primary vaccination [32]. Furthermore, it was observed that dogs with expired vaccination status exhibited an antibody response to booster rabies vaccination comparable to that of dogs with up-to-date vaccination status [44]. Given the fast turnover of dog populations in rabies-endemic countries, this generic approach could be adaptable for use in these regions. Our assumption of constant mortality rate may lead to overestimate the proportion of dogs with protective immunity. While the current approach, which overlooks age-related variations in mortality rates and rates of loss of protective immunity, is preliminary and in need of refinement through extended follow-up, its genericity makes it potentially applicable to other rabies-endemic countries with rapid dog population turnover.

Based on the declining seropositivity rate after one year, booster vaccinations of dogs are recommended [13]. Dogs that are primary vaccinated when < six months should receive a booster dose not later than one year following administration of the first dose [6]. Annual parenteral vaccination of ≥70% of the dog population is effective to control rabies but difficult to achieve in LMICs due to lack of adequate resources, and aggressive and/or free-roaming dogs. Several surveys from focal point vaccination campaigns showed that 15–40% of adult dogs were not brought to veterinarians because they were hard to handle [45–47]. The above-mentioned constraints may result in a declined vaccination coverage to an insufficient 20–45% [27].Very few surveys have been carried out under field conditions, based on immunity duration and taking into account dog population dynamic to propose pragmatic vaccination strategies. A study in Kinshasa, where the dog population characteristics are close Cambodia’s dog population, demonstrated that systematic vaccination of puppies as well as annual vaccination of dogs aged between 3 and 15 months would be an efficient alternative to annual MDV [48]. In Cambodia, most dogs are owned but are also free roaming and are therefore often hard to handle. In contrast, younger dogs are more reachable since they mostly stay around their owner’s houses and are easier to handle. Here, we show that the proportion of protected dogs would reach 83% in Kandal and 85% in Battambang with an annual vaccination of <1 year old dogs, and a systematic booster injection of dogs that had been vaccinated the year before. This strategy would allow controlling rabies transmission in both investigated dog populations within 3–5 years. We estimated 14–34% of vaccine doses required for annual vaccination of all dogs could be saved by implementation of strategy B. Furthermore, not only vaccine doses would be saved but also personnel costs would be reduced with strategy B as less dogs need to be vaccinated and younger dogs (<12 months) are usually easier to handle than adult dogs.

The here described model is the first one outlining the life-long immunity induced in dogs after only two vaccinations and its implications for rabies control. These results can contribute to adapt control measures in Cambodia, but also in other countries with similar dog population characteristics moving towards the global goal of ending human deaths from dog-mediated rabies by 2030.

## Supporting information

S1 Text R and Stan codes, and anonymized raw data. “dogs_model” was used to estimate parameters and compare different vaccination strategies, using the following stan programs: "dogs_H0.stan" file to test the null hypothesis of an absence of link between the model parameters and individual covariables, and “dogs_H1_pSe.stan”, “dogs_H1_pSV.stan”, “dogs_H1_rBS.stan”, “dogs_H1_rVS.stan”, “dogs_H1_rFOI.stan” and “dogs_H1_rFOI_cage.stan” files to test alternative assumptions.
(ZIP)

S2 Text Evaluation of vaccination strategies–Calculations. (DOCX)
