# Supplementary material for: Bayesian modeling of post-vaccination serological data suggests that yearly vaccination of dog aged <2 years old is efficient to stop rabies circulation in Cambodia
Source: PLoS Negl Trop Dis. 2024 Apr 18;18(4):e0012089. doi: 10.1371/journal.pntd.0012089 (PMC11060556; doi:10.1371/journal.pntd.0012089)
Supplement: S2 Text — (DOCX) [file pntd.0012089.s002.docx]

**Supplementary material 2**

## Evaluation of vaccination strategies – Calculations

Assuming that the implementation of the strategy takes place on a fixed date each year, the proportion of protected dogs after the vaccination in year *N* was calculated using the following formula:

$${\sum_{y=0}^{N} Imm(y)}/{\sum_{y=0}^{N} Surv(y)}$$

where *Surv(y)* denotes the proportion of alive dogs among those born *y* years earlier

$$Surv\left( y \right)=e^{-\mu12y}$$

(with *µ* the monthly mortality rate, assumed not to vary with age). In the numerator, $Imm(y)$ indicates the proportion of live and protected dogs among those *y* years old. This proportion is identical in both strategies for <1 year old dogs with$Imm\left( y \right)=q$, i.e., the proportion of young dogs in which primary vaccination induced a protective immunity (we neglected the mortality between the date of vaccine injection and the date at which the proportion was calculated). For dogs ≥1 year old, $Imm(y)$ varied according to the strategy.

For strategy A,

$$Imm\left( y \right)=q e^{-12y\left( \mu+\rho_{1} \right)}$$

i.e., the proportion of dogs that developed a protective immunity upon vaccination, and have not died, and have not lost their protective immunity since vaccination (the monthly rate of immunity loss being *ρ*_1_).

For strategy B,

$$Imm\left( y \right)={Imm}_{a}\left( y \right)+{Imm}_{b}(y)$$

as we distinguished two types of dogs.

First,dogs in which the first vaccine injection induced protective immunity until the booster injection:

$${Imm}_{a}\left( y \right)=q e^{-12\left( \mu+\rho_{1} \right)}e^{-12(y-1)\left( \mu+\rho_{2} \right)}$$

with *q*the probability that the primary vaccination induced protective immunity, $e^{-12\left( \mu+\rho_{1} \right)}$ the probability that the dog survived with protective immunity until the booster vaccination, and $e^{-12(y-1)\left( \mu+\rho_{2} \right)}$ the probability that the protective immunity induced by the booster vaccination had not been lost since then.

Second, dogs in which the primary vaccination did not induce a protective immunity, or who lost their protective immunity before the booster vaccination:

$${Imm}_{b}\left( y \right)=\left[ \left( 1-q \right) e^{-12\mu}+q e^{-12\mu} \left( 1-e^{-12\rho_{1}} \right) \right] qe^{-12\left( y-1 \right)\left( \mu+\rho_{1} \right)}$$

with $\left( 1-q \right) e^{-12\mu}$ the probability that the primary vaccination did not induce immunity with survival of the dog until the booster injection, and $q e^{-12\mu} \left( 1-e^{-12\rho_{1}} \right)$ the probability that, the primary vaccination induced protective immunity and the dog survived, but lost its immunity before the booster vaccination. For this category of dogs, we simplified and assumed the booster vaccination had the same effect as a primary vaccination; $qe^{-12(y-1)(\mu+\rho_{1})}$ as the probability that the dog had acquired protective immunity after the booster vaccination and had maintained it since then.
